# Serum concentrations of haptoglobin and haptoglobin-matrix metalloproteinase 9 (Hp-MMP 9) complexes of bovine calves in a bacterial respiratory challenge model

**DOI:** 10.1186/s12917-014-0285-5

**Published:** 2014-12-06

**Authors:** Christy J Hanthorn, Grant A Dewell, Renee D Dewell, Vickie L Cooper, Chong Wang, Paul J Plummer, Jeffrey Lakritz

**Affiliations:** Department of Veterinary and Diagnostic Production Animal Medicine, College of Veterinary Medicine, Iowa State University, Ames, IA 50011 USA; Department of Veterinary Microbiology and Preventive Medicine, Center for Food Security and Public Health, College of Veterinary Medicine, Iowa State University, Ames, IA 50011 USA; Department of Veterinary Microbiology and Preventive Medicine, College of Veterinary Medicine, Iowa State University, Ames, IA 50011 USA; Department of Statistics, College of Liberal Arts and Sciences, Iowa State University, Ames, IA 50011 USA; Department of Veterinary Clinical Sciences, College of Veterinary Medicine, The Ohio State University, Columbus, OH 43210 USA

**Keywords:** Hp-MMP 9, Haptoglobin, MMP 9, Bovine Respiratory Disease, Pneumonia, Calf

## Abstract

**Background:**

Serum haptoglobin (Hp) and haptoglobin matrix metalloproteinase 9 complexes (Hp-MMP 9) have been identified as biomarkers with diagnostic potential in cattle with conditions resulting in an acute inflammatory response. The purpose of this study was to evaluate potential diagnostic applications of serum Hp and Hp-MMP 9 concentrations in calves with BRD and establish a timeline for their detection in calves experimentally challenged with *Bibersteinia trehalosi* and *Mannheimia haemolytica.* Thirty-five cross bred dairy calves were inoculated via tracheal catheterization with either a PCR confirmed leukotoxin negative *B. trehalosi* isolate, a PCR confirmed leukotoxin positive *B. trehalosi* isolate, a *Mannheimia haemolytica* isolate, a combination of leukotoxin negative *B. trehalosi* and *M. haemolytica*, or a negative control. Serum samples were collected throughout the study. Calves were euthanized and necropsy performed on day 10 post inoculation.

**Results:**

*M. haemolytica* inoculated calves had increased lung involvement. Serum Hp and Hp- MMP 9 concentrations were elevated compared to the other treatment groups. Increases in serum Hp and Hp-MMP 9 concentrations for the *M. haemolytica* group were significantly different from other study groups on day 7 of the study. *B. trehalosi* inoculated calves did not have increased lung involvement compared to control calves, but the leukotoxin positive *B. trehalosi* group demonstrated increased serum Hp-MMP 9 concentrations from day 3 to the end of the study compared to the pre-inoculation concentrations.

**Conclusion:**

Serum Hp-MMP 9 concentration is a useful diagnostic tool for detecting early pulmonary inflammation in calves challenged with *B. trehalosi* and *M. haemolytica*. Serum Hp-MMP 9 may also be a useful tool in detecting subclinical pulmonary inflammation in challenged calves.

## Background

Bovine respiratory disease (BRD) has been shown to affect various biomarkers in cattle [1-7]. Two biomarkers that have been identified for diagnostic potential are serum haptoglobin (Hp), matrix metalloproteinase 9 (MMP 9), and the complexes that they are able to form (Hp-MMP 9) [1,6-9].

Free Hp is an alpha - 2 globulin that is primarily synthesized in the liver [3]. Free Hp’s primary function is to bind to free hemoglobin in the blood. By scavenging the free hemoglobin, Hp helps to prevent oxidative tissue damage and conserve iron by returning the heme residue to the host’s metabolic process. This scavenging mechanism also serves to decrease the risk of free hemoglobin being utilized for bacterial pathogen growth [7,8].

MMP 9 is a collagenase belonging to the gelatinase B group [5]. The gelatinase B group is a family of MMP’s that are zinc dependent proteinases capable of degrading at least one component of the extracellular matrix or basement membrane. This capability assists in the migration of white blood cells, mostly neutrophils, from the blood to the site of inflammation [4]. MMP 9 is stored in tertiary granules within the bovine neutrophils and is released when neutrophil degranulation is induced by either chemical or microbial stimuli [1,5,10,11]. After release, MMP 9 is able to cleave interleukin 8, if present in the microenvironment into active interleukin 8. This creates a positive feedback loop for neutrophil migration [10]. While only neutrophils have been demonstrated to store MMP 9 for immediate release [8], other cells such as alveolar macrophages can be induced to produce MMP 9 [5,12].

Free Hp and MMP 9 are secreted by multiple cellular sources in response to a variety of challenges; Hp-MMP 9 complexes have been shown to form exclusively in neutrophils [1]. These complexes are stored until neutrophil degranulation at which time they are released [1,6,8]. Hp has been demonstrated to have a high diagnostic sensitivity for detecting infectious or inflammatory conditions. Due to its poor specificity, it cannot be used as the primary diagnostic test for any one condition. Free Hp is also poor at differentiating between acute and chronic inflammation [1,7]. Free MMP 9 is more specific to acute inflammation, but it is not specific enough to differentiate between healthy and diseased cattle [1,4]. Hp-MMP 9 complexes are capable of differentiating between acute and chronic inflammation as their release from neutrophils is associated with acute inflammatory responses [1,8].

Various biomarkers potentially associated with BRD have been studied. In previous studies Hp was demonstrated to increase after calves were exposed to *Mannheimia haemolytica* via intra-tracheal inoculation with the earliest detection occurring at 24 hours, and peak concentrations occurring at 3 days post inoculation [6,7]. In another challenge study, increases in serum Hp concentrations in calves infected with BVDV were not detected until 7–9 days post infection [7]. Serum concentrations of Hp-MMP 9 have not been evaluated in a BRD challenge study. The purpose of this study was two-fold: to evaluate the diagnostic applications of serum Hp-MMP 9 concentrations in calves with BRD and to establish a timeline for their detection in calves undergoing experimental challenge with *Bibersteinia trehalosi* and *Mannheimia haemolytica.*

## Methods

Prior to initiating the study, the protocol was approved by the Iowa State University Institutional Animal Care and Use Committee (IACUC 8-11-7187-B) and the Institutional Biosafety Committee (IBC#11-D-0017-A). This study is a hypothesis generating study representing a secondary use of calves enrolled in a challenge study designed to evaluate the pathogenicity of *Bibersteinia trehalosi* in respiratory disease among bovine calves [13]. The rationale for the secondary use of these calves to meet additional objectives is consistent with using the 3R principles to maximize information obtained from animal research [14].

Individually housed calves were inoculated via tracheal catheterization with of either a PCR (lktA) confirmed leukotoxin negative *B. trehalosi* isolate (8 calves), a PCR confirmed leukotoxin positive *B. trehalosi* isolate (8 calves), a *Mannheimia haemolytica* isolate (7 calves), a combination of leukotoxin negative *B. trehalosi* and *M. haemolytica* (8 calves), or a negative control (4 calves) as previously described [13]. The B. trehalosi isolates were field strains obtained from diagnostic submissions and the *M. haemolytica* isolate was a proprietary leukotoxin positive isolate. Calves were inoculated with 20 ml of a Brain Heart Infusion broth containing approximately 2.5 × 10^9^ CFU of bacterial per milliliter.

Blood samples were collected from each calf via jugular venipuncture on Days 1 (pre-inoculation), 3, 5, 7, 9, and 10 (immediately prior to euthanasia). Blood samples were centrifuged at 4000 rpm for 15 minutes. Following centrifugation, samples were placed on ice before being transferred, using a transfer pipette, to an appropriately labeled cryovial. Boxes containing cryovials were placed immediately in an ultra-low freezer and stored at - 70°C until being shipped to The Ohio State University on dry ice for analysis.

All surviving calves were euthanized on day 10 of the study, necropsied, and evaluated for percent abnormal lung involvement as previously described [13].

### Serum Haptoglobin-Matrix metalloproteinase 9 (Hp-MMP 9) ELISA assay

Bovine Hp-MMP 9 complexes were determined as described previously [1,6,15]. All serum samples were diluted 1:5 with sample diluent (TBS +1% Bovine serum albumin +0.05% Tween 20). After blocking the wells (4°C for 120 minutes), known concentrations of Hp-MMP 9 (serum, pre-characterized and shown to contain ~912.6 ng/mL Hp-MMP 9) and the challenged calf serum samples were added to wells. Serum from healthy cows was used as a negative control.

### Serum total haptoglobin ELISA assay

Serum Hp concentrations were determined as described (Bovine haptoglobin 96-well ELISA. Life Diagnostics, West Chester, PA 19380) using commercial Bovine haptoglobin ELISA test kits, according to manufacturer’s instructions. Standard curves were prepared using purified bovine haptoglobin standard (2.5 μg/mL) included with the kit at a concentration range from 7.8 – 250 ng/mL. Serum samples were diluted according to the kit instructions (1:2,000 dilution) and were run in duplicate. Controls included were normal bovine serum, 5% BSA in TBS and blank wells. Linear regression of the Hp calibrator concentration versus absorbance was used to determine the equation for the line. The slope and intercept of this line was used to calculate the concentration of serum total Hp in the unknown animal samples. These concentrations were corrected for the dilution factor (× 2,000) and the concentrations reported in μg/mL.

### Statistical analysis

Hp- MMP 9 and Hp were analyzed by repeated measures analysis of variance (ANOVA), with treatment, time, and their interaction as fixed effects and animal as subject of repeated measures. Lung involvement was analyzed by analysis of variance model, with treatment as explanatory variable. Correlations were calculated among the values of lung involvement, Hp, and Hp-MMP 9 for both Hp and Hp-MMP 9 serum concentrations as both ranked and absolute values prior to the challenge as well as averaged over the time of the study. SAS® Version 9.2 (SAS® Institute Inc., Cary, NC, USA) was used in analyses. A p-value <0.05 was considered significant.

## Results and discussion

### Lung involvement

Prior to inoculation all calves appeared clinically normal. *Pasteurella multocida* was isolated from pharyngeal swab samples from 34 of 35 calves. *M. haemolytica* was isolated from 8 of 35 calves. *M. haemolytica* positive pharyngeal swab calves were spread across all five treatment groups (1/4 in control group, 1/8 in leukotoxin negative *B. trehalosi* group, 2/8 in leukotoxin positive *B. trehalosi* group, 1/7 in *M. haemolytica* group, 3/8 in leukotoxin negative *B. trehalosi* and *M. haemolytica* combination group).

Six of the 35 enrolled calves were euthanized prior to day 9 of the study. Three calves (3/7) from the *M. haemolytica* group (days 2, 3 and 3) and three calves (3/8) from the leukotoxin negative *B. trehalosi* and *M. haemolytica* combination treatment (days 3, 5 and 8) were euthanized according to protocol because of a clinical assessment of moribund.

**Figure 1:**
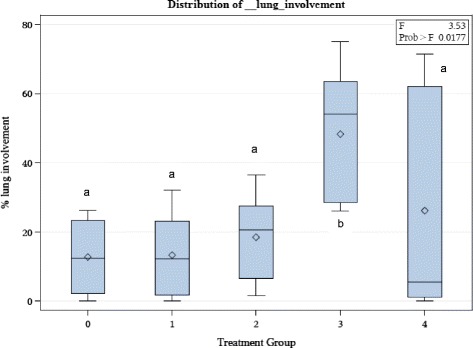
**Distribution of lung involvement.** Distribution of percent lung involvement by treatment group. Treatment group 0: Negative control group, Treatment group 1: leukotoxin negative *B. trehalosi* group, Treatment group 2: leukotoxin positive *B. trehalosi* group, Treatment group 3: *M. haemolytica* group, Treatment group 4: Combination of leukotoxin negative *B. trehalosi* and *M. haemolytica* group. Means with different letters are significantly different (p < 0.05). Diamonds represent the group mean. Boxes represent the middle two quartiles of individual values, divided by the center line which represents the median. Bars adjacent to the boxes represent the outer two quartiles of individual values.

The mean estimated percent lung involvement was highest for the *M. haemolytica* group (49%). The mixed infection group of leukotoxin negative *B. trehalosi* and *M. haemolytica* had a mean lung involvement of 26%. The leukotoxin positive *B. trehalosi* had a mean lung involvement of 18% while the leukotoxin negative *B. trehalosi* mean lung involvement was estimated to be 13%. The control group had a mean lung involvement estimate of 13%. There was evidence of a statistically significant (p = 0.018) difference for mean percent total lung involvement between the *M. haemolytica* group and the leukotoxin negative *B. trehalosi* group. There were no significant differences between the other treatment groups. Even though the control group had a lower mean percent lung involvement than the leukotoxin negative *B. trehalosi* treatment group, the low number (4 calves) of study subjects in the control group did not have sufficient power to make it significantly different from the *M. haemolytica* treatment group. The mean and median values were similar in all treatment groups except the mixed infection group. This group had a high amount of variability within it as evidenced by a mean percent lung involvement of 26% and a median percent lung involvement of 5%. The high variability made drawing conclusions difficult in this group (Figure 1).

### Free Hp

Other studies have found that serum Hp is nearly undetectable in healthy cattle [8]. Concentrations up to about 200 μg/mL are considered acceptable for healthy animals [2,7,9,16]. Concentrations between about 200 and 400 μg/mL are associated with mild inflammation and concentrations greater than about 400 μg/mL suggest severe inflammation [7]. Serum Hp concentration does not differ with age or sex in cattle [2,7,9].

In this study, 11 calves (2/4 in the control group, 2/8 in the leukotoxin negative *B. trehalosi* group, 4/8 in the leukotoxin positive *B. trehalosi* group, 1/7 in the *M. haemolytica* group, and 2/8 in the leukotoxin negative *B. trehalosi* and *M. haemolytica* combination group) had serum Hp concentrations greater than 200 μg/mL prior to inoculation. Two of the calves had low serum concentrations of Hp-MMP 9 indicating the presence of chronic inflammation. *M. haemolytica* was cultured from the pharyngeal swabs of both calves. Nine of the calves with high serum concentrations of Hp also had high serum concentrations of Hp-MMP 9 indicating the presence of chronic inflammation which was associated with an acute inflammatory event. Three of these calves were culture positive for *M. haemolytica* from pharyngeal swabs. Nineteen calves had no detectible serum Hp prior to inoculation.

**Figure 2:**
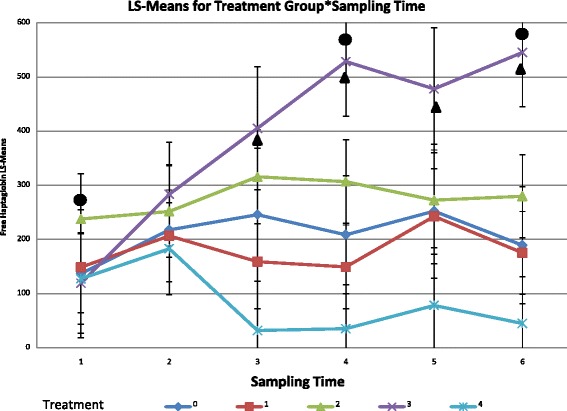
**Means of serum HP concentration for treatment group by date.** Serum Hp concentration in micrograms/mL over time by treatment group. Treatment group 0: Negative control group, Treatment group 1: leukotoxin negative *B. trehalosi* group, Treatment group 2: leukotoxin positive *B. trehalosi* group, Treatment group 3: *M. haemolytica* group, Treatment group 4: Combination of leukotoxin negative *B. trehalosi* and *M. haemolytica* group. Circles above trend lines indicate significant differences from other treatment groups. Triangles below trend lines indicate significant differences from prior bleeding dates.

The interaction between treatment group and bleeding date was a statistically significant effect (p = 0.01). The mean serum Hp concentration for the leukotoxin positive *B. trehalosi* treatment group was significantly (p = 0.008) different from the other treatment groups for the pre-inoculation bleeding date, indicating that more calves with evidence of chronic inflammation prior to commencement of the study were randomly assigned to this treatment group. On the fourth and sixth bleeding dates (days 7 and 10) there was a significant (p < 0.05) difference in the mean Hp concentration between the *M. haemolytica* treatment group and the leukotoxin negative *B. trehalosi* treatment group and the leukotoxin negative *B. trehalosi* and *M. haemolytica* combination group. The *M. haemolytica* treatment group demonstrated significant elevations in serum Hp concentrations from the first two bleeding dates to the third bleeding date (day 5) (p = 0.02). The elevation in serum Hp concentration remained significant (p < 0.02) throughout the study. This finding is in agreement with other studies that have demonstrated an increase in serum Hp after *M. haemolytica* infection [4,6]. Concentrations in this study peaked on day 7 rather than day 3 as previously reported. A decrease in the serum Hp concentration for the mixed infection group can be observed between the second and third bleeding dates (days 3 and 5). This observation supports findings by Dassanayake et al. suggesting that *B. trehalosi* is capable of inhibiting the growth of *M. haemolytica* in vivo [17]. In this study the difference in serum Hp concentration is not significantly different from the other treatment groups. Two calves from this treatment group were euthanized before the third bleeding time point. The smaller treatment group likely did not have enough power for a statistical difference to be observed (Figure 2).

### Hp-MMP 9

The mean serum Hp-MMP 9 concentration for all treatment groups prior to inoculation was 104 ng/mL, with a minimum value of 0 ng/mL, a maximum value of 752.2 ng/mL, and a median value of 4.15 ng/mL. Little to no Hp-MMP 9 should be detectible in the serum of healthy calves [6]. Sixteen calves had no detectible serum Hp-MMP 9 concentration prior to inoculation. For this study a serum concentration greater than 20 ng/mL was considered high. Thirteen calves had high serum Hp-MMP 9 concentrations prior to inoculation. Four of the thirteen calves had low serum concentrations of Hp, suggesting the presence of a mild acute inflammatory process. None of these 4 calves were culture positive for *M. haemolytica* from the pharyngeal swabs. Nine of the thirteen calves had high serum concentrations of Hp and were discussed previously.

**Figure 3:**
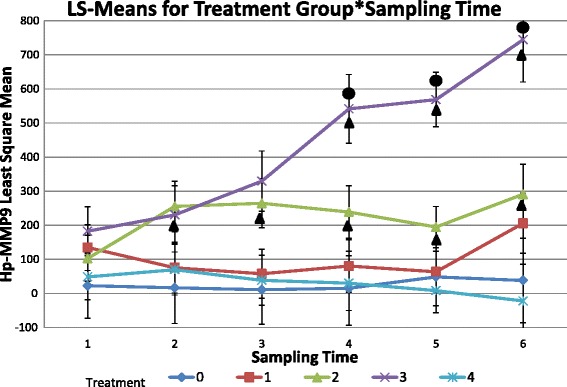
**Means of serum Hp-MMP 9 concentration for treatment group by date.** Serum Hp-MMP 9 concentration in nanograms/mL over time by treatment group. Treatment group 0: Negative control group, Treatment group 1: leukotoxin negative *B. trehalosi* group, Treatment group 2: leukotoxin positive *B. trehalosi* group, Treatment group 3: *M. haemolytica* group, Treatment group 4: Combination of leukotoxin negative *B. trehalosi* and *M. haemolytica* group. Circles above trend lines indicate significant differences from other treatment groups. Triangles below trend lines indicate significant differences from prior bleeding dates.

Treatment group, bleeding date, and their interaction were all significant (p < 0.01) effects. On the fourth bleeding date (day 7) the mean serum Hp-MMP 9 concentration for the *M. haemolytica* group became significantly different from the negative control, the leukotoxin negative *B. trehalosi*, and the leukotoxin negative *B. trehalosi* and *M. haemolytica* combination groups (p = 0.01, p = 0.009, and p = 0.004 respectively). This difference continued throughout the study. On the fifth bleeding date (day 9) the mean serum Hp-MMP 9 concentration for the *M. haemolytica* group became significantly different from the leukotoxin positive *B. trehalosi* group (p = 0.006) and remained different throughout the rest of the study. The *M. haemolytica* group demonstrated significant elevations in serum Hp-MMP 9 concentrations from the first three bleeding dates to the last three bleeding dates (p < 0.02). The leukotoxin positive *B. trehalosi* treatment group demonstrated a small but significant (p < 0.004) elevation in serum Hp-MMP 9 concentrations from the pre-inoculation bleeding date to the second bleeding date (day 3) that continued throughout the study (Figure 3). This finding is consistent with other studies that have demonstrated the rapid recruitment and accumulation of neutrophils at the onset of BRD [6] as well as the action of leukotoxins to stimulate the active degranulation of bovine neutrophils [5].

### Correlations

There was statistically significant (p < 0.0001) evidence of a strong (r = 0.7) correlation between Hp and Hp-MMP 9 serum concentrations as both ranked and absolute values prior to inoculation. Evidence of a relationship remained statistically significant over time (p < 0.0001), but the strength of the relationship varied depending on if a Pearson (absolute values) or Spearman (ranked data) correlation coefficient was calculated. The Pearson correlation coefficient was r = 0.63; whereas, the Spearman correlation coefficient was r = 0.79. This strong relationship was expected since both substances have been established as biomarkers for inflammation and the test for free Hp measures Hp-MMP 9 contribution also.

There was statistically significant evidence (p < 0.05) of a moderate correlation (r = 0.44 and r = 0.35) that became stronger over time (r = 0.67 and r = 0.48) between Hp-MMP 9 and percent lung involvement for both ranked and absolute values respectively. There was statistically significant evidence (p = 0.04) of a moderate (r = 0.35) correlation between free Hp and percent lung involvement for ranked, but not absolute values prior to inoculation. This correlation became stronger (r = 0.54, p = 0.0008) when values were averaged over the time of the study. The correlation between free Hp and percent lung involvement for absolute data was also statistically significant when averaged over time (r = 0.41, p = 0.015).

This correlation data indicates that Hp-MMP 9 serum concentrations may be a good ante-mortem diagnostic indicator of lung damage that is found at the time of the calf’s death. This data also supports the conclusion from other studies that Hp-MMP 9 serum concentration is a better diagnostic test for lung damage than free Hp serum concentration [1]. Future studies should be conducted to determine reference ranges of serum Hp-MMP 9 for different levels of disease severity.

## Conclusion

All study calves appeared to be clinically normal prior to inoculation in this study. Culture results of pharyngeal swabs taken from calves prior to inoculation showed that all but one calf was positive for *P. multocida*. The presence of this bacterium did not appear to have an impact on pre-inoculation values of serum Hp or Hp-MMP 9 since many of the calves had serum concentrations of these two biomarkers that were within limits considered acceptable for healthy animals [2,7,9,16]. The pharyngeal swabs for 8 calves were culture positive for *M. haemolytica* prior to inoculation. This finding could have potentially affected the development of lung lesions as well as increases in serum Hp and Hp-MMP 9 concentrations in calves that were members of treatment groups other than the *M. haemolytica* group. The effect was not large enough to preclude seeing statistically significant results from the challenge in the *M. haemolytica* group. The development of a rapid, inexpensive test for serum Hp-MMP 9 concentrations could have clinical applications as a screening test for subject enrollment in BRD studies. Subjects with elevated serum concentrations could be excluded from participation.

Significant differences were observed between the *M. haemolytica* group and the other treatment groups in percent total lung involvement, serum Hp, and serum Hp-MMP 9 concentrations. The difference in percent total lung involvement demonstrates that the inoculation technique was appropriate for this challenge model. The increase in serum Hp and Hp-MMP 9 concentrations at day 7 are consistent with other reports of these two biomarkers having diagnostic potential with respect to BRD. Results suggest a different, slightly later, timeframe for using them as diagnostic tools.

No significant difference was observed between the leukotoxin positive *B. trehalosi* treatment group and the negative control, leukotoxin negative *B. trehalosi*, and combination of leukotoxin negative *B. trehalosi* and *M. haemolytica* groups with respect to percentage of total lung involvement or serum Hp concentrations. A small but significant increase was noted in the serum Hp-MMP 9 concentrations of the leukotoxin positive *B. trehalosi* treatment group from day 1 to day 3, indicating that serum Hp-MMP 9 concentrations may be a more sensitive test for pulmonary inflammation than visible lung damage or serum Hp concentrations. This finding also provides evidence that the leukotoxin positive strain of *B. trehalosi* induced more neutrophil degranulation leading to a higher release of Hp-MMP 9 complexes than the leukotoxin negative strain of *B. trehalosi*. In this study the earlier rise in the serum Hp-MMP 9 concentration for the leukotoxin positive *B. trehalosi* treatment group than for the *M. haemolytica* treatment group may indicate that bovine neutrophils respond faster to a *B. trehalosi* infection than to a *M. haemolytica* infection.

The stronger significant correlation between serum Hp-MMP 9 concentration and percent lung involvement than between serum Hp concentration and percent lung involvement support the conclusions of other studies that serum Hp-MMP 9 concentrations can be an effective tool for early diagnosis of BRD [6] and that it is a better diagnostic tool for acute inflammation than serum Hp [1]. Future studies to determine a reference range for serum Hp-MMP 9 concentration and to develop a rapid, inexpensive test would help to make serum Hp-MMP 9 concentration a clinically useful diagnostic tool.

## Abbreviations

Hp-MMP 9: Haptoglobin-matrix metalloproteinase 9
Hp: Haptoglobin
MMP 9: Matrix metalloproteinase 9
BRD: Bovine respiratory disease
BVDV: Bovine viral diarrhea virus
PI: Persistently infected
PCR: Polymerase chain reaction
*M. haemolytica*: *Mannheimia haemolytica*
*B. trehalosi*: *Bibersteinia trehalosi*
*P. multocida*: *Pasteurella multocida*
